# Re-Assemblable, Recyclable, and Self-Healing Epoxy Resin Adhesive Based on Dynamic Boronic Esters

**DOI:** 10.3390/polym15163488

**Published:** 2023-08-21

**Authors:** Zhiyong Liu, Zhiguo Song, Benrong Lv, Zumin Qiu

**Affiliations:** 1 Huzhou Guoneng New Material Co., Ltd., Huzhou 313000, China; 2 School of Chemistry and Chemical Engineering, Nanchang University, Nanchang 330031, China; 3 Department of Polymer Materials and Engineering, School of Chemical and Environmental Engineering, Anhui Polytechnic University, Wuhu 241000, China

**Keywords:** biomass, epoxy resin adhesive, dynamic covalent bond, boronic ester, re-assemble

## Abstract

Thermosetting adhesives are commonly utilized in various applications. However, covalent cross-linked networks prevent thermosetting adhesives from being re-assembled, which necessitates higher machining precision. Additionally, the primary raw materials used in adhesive preparation are derived from non-renewable petroleum resources, which further constrain adhesive development. In this study, a recyclable adhesive was developed by incorporating dynamic boronic esters into epoxy resin derived from soybean oil. The successful synthesis of epoxidized soybean oil and boronic esters was confirmed through the analysis of proton nuclear magnetic resonance spectra and differential scanning calorimetry results. Swelling tests and tensile curves demonstrated the presence of covalently cross-linked networks. Self-healing and reprocessing experiments indicated that the cross-linked network topology could be re-assembled under mild conditions.

## 1. Introduction

Adhesives are used to connect different types of materials through adhesion and cohesion at their interfaces [1,2,3,4,5]. They have a wide range of applications in various fields, including aerospace, transportation, instruments, electronic appliances, machinery manufacturing, and daily necessities [6,7,8,9,10,11]. Epoxy resin is a very important type of adhesive. Its performance can be altered through chemical structural design to meet different requirements in various application scenarios. There are mainly two methods for synthesizing epoxy resin: one method involves the condensation of polyols, polyacids, or polyamines with compounds containing epoxy groups. The other involves the epoxy oxidation of linear or cyclic diene compounds with peroxides. The reaction conditions differ depending on the type of double bond. When the double bond exists in an aliphatic chain, its epoxy oxidation is very simple and only requires hydrogen peroxide.

Epoxy resin is mostly in a high viscosity liquid state before reacting with a curing agent and has no practical value. At this time, low molecular weight polymers are commonly referred to as epoxy oligomers. After reacting with an acid anhydride or polyamine curing agent to form cross-linked networks, epoxy resin exhibits excellent adhesion, thermal stability, chemical corrosion resistance, and high mechanical strength. Therefore, it is widely used in important industrial fields such as automotives, shipbuilding, aerospace, and electronic packaging. However, due to the covalent cross-linking forming irreversible cross-linked networks, epoxy resin often exhibits drawbacks such as high brittleness, poor impact resistance, and low toughness. When subjected to external impact, it is prone to damage, leading to the formation of many micro-cracks within the bulk, which affects the mechanical stability and service life of epoxy resin. Additionally, like other thermosetting adhesives, the irreversible covalent cross-linked networks make epoxy resin non-melting and non-dissolvable once it is formed, preventing it from being disassembled and recycled as needed. As a basic industrial material, epoxy resin is widely used, and its production is increasing year by year. If epoxy resin cannot be repaired or recycled in a timely manner, it will result in a large amount of waste, leading to serious resource waste and environmental pollution.

In order to overcome the defects of thermosetting epoxy resin in a reasonable manner, the incorporation of dynamic covalent bonds to construct covalent adaptable networks (CANs) in epoxy resin to achieve a re-assemblable, recyclable, and self-healing epoxy resin adhesive is an effective strategy, garnering considerable attention in recent years [12,13,14,15,16,17,18,19,20]. This concept was first proposed by Bowman and coworkers [21]. The reported networks included a certain number and topological structure of dynamic covalent bonds, allowing the cross-linked network structure to respond chemically to applied stimuli (such as heat, light, or pH) and achieve network reorganization. The construction of CANs bridges the gap between thermosetting resins and thermoplastic resins. On the one hand, it allows highly cross-linked thermosetting resins to “flow” like thermoplastics at high temperatures. On the other hand, it is expected to provide good recyclability and self-healing properties through chain rearrangement, while maintaining dimensional stability, insolubility, and non-melting characteristics at room temperature. It should be noted that although CANs, like thermoplastic resins, possess recyclability, they are fundamentally different. The recyclability of thermoplastic resins comes from the slip between polymer chains, while the recyclability of CANs is ascribed to the exchange of dynamic covalent bonds. Under certain conditions, if there is internal stress in the polymer network, the topological structure of the network can be rearranged through bond exchange behavior to achieve stress release, which is macroscopically manifested as stress relaxation of the polymer. This also gives the system the characteristic of recyclability.

Thermosetting resins based on dynamic covalent bonds, through molecular structure design, have simple recycling processes and do not generate by-products, thus greatly extending the service life of resins and improving resource utilization. In 2011, Leibler et al. proposed the concept of vitrimers, in which zinc acetate was added as a catalyst to the curing system of bisphenol A epoxy resin and aliphatic carboxylic acid/anhydride, obtaining a polymer based on dynamic ester bonds [22]. Rheological tests showed that the obtained polymer exhibited rheological properties similar to glass. At room temperature, it exhibited similar characteristics to traditional thermosetting resins, but as the temperature increased, its viscosity gradually decreased. During processing, even if the local temperature decreases, it does not immediately stop flowing, with it maintaining structural integrity within a certain temperature range. However, vitrimers based on transesterification often require catalysts containing metal ions, which can reduce material compatibility, accelerate material aging, and affect its performance stability. To improve this issue, catalyst-free dynamic covalent bonds such as imines, transamination, and boronic esters have been developed [23,24,25]. Boronic esters are formed by the complexation of phenylboronic acid and diols. In aqueous solutions, the strength of boronic esters depends on the pH of the solution and the pKa of phenylboronic acid. When the pH of the solution is higher than the pKa of phenylboronicic acid, boronic esters are easily formed. Conversely, when the pH of the solution is lower than the pKa of phenylboronicic acid, the equilibrium tends towards free phenylboronicic acid and diols. Due to the dynamic equilibrium between phenylboronic acid–diols and boronic esters, the exchange of esters leads to self-healing when the boronic esters are at the damaged interface of the material. Additionally, under solvent-free conditions, the exchange reactions can also occur between boronic esters, allowing for network rearrangement. Many researchers have conducted in-depth research on boronic esters. For example, Guan et al. revealed the principle that the difference in electronegativity of substituents at the ortho position can lead to differences in the kinetics of exchange reactions by studying the rate of boronic ester exchange reactions [26]. The authors cross-linked polymers with two different boronic esters and studied the effect of the rate of exchange reactions on polymer network rearrangement. The results showed that polymer systems with faster boronic ester exchange reaction rates exhibited excellent self-healing performance and good recyclability, with the mechanical properties of the material remaining almost unchanged after three cycles of recycling. In contrast, polymer systems with slower boronic ester exchange rates had almost no self-healing ability and poor ability for recycling.

Moreover, with the increasing depletion of non-renewable resources such as petroleum and growing environmental consciousness among individuals, global politics and institutions have shifted towards principles of sustainable development. This has prompted the chemical industry to focus on sustainable chemistry, particularly in the production of high-performance materials using biomass molecules. Biomass molecules possess renewable, inexhaustible, and abundant characteristics, making them a promising market for the future. Among biomass molecules, plant-derived vegetable oils, rich in triglycerides, are currently the most widely used renewable resources in the chemical industry [27]. Soybean oil, castor oil, palm oil, and rapeseed oil are extensively employed as primary raw materials for the synthesis of various bio-based products. Currently, numerous researchers have reported the chemical modification and conversion of triglycerides into polymerizable monomers through epoxidation. Among these, the derivatives of soybean oil (SBO) are favored due to its abundant availability [28,29]. Triglycerides typically possess three ester bonds, which can be hydrolyzed to form glycerol and three fatty acids. Fatty acids constitute approximately 95% of the total weight of triglycerides. Different plant oils exhibit varying compositions of fatty acids, with most fatty acids possessing natural functionalities such as double bonds and hydroxyl groups. In light of the depletion of global fossil resources, there is a significant imperative to explore the utilization of renewable biomass resources in the production of adhesives [30,31,32,33,34,35,36,37].

In this paper, soybean oil (SBO) was utilized as the initial polymer substrate and subsequently functionalized by incorporating epoxy groups. The resultant epoxy resin adhesive was formulated to possess self-healing properties and recyclability by incorporating SBO and boronic esters. The chemical structures of the epoxidized SBO and the synthesized cross-linker were meticulously characterized. Subsequently, various compositions of cross-linked epoxy resins were prepared by reacting the respective groups. The findings of this study demonstrate the re-assembly capability of the epoxy resin adhesive at a moderate temperature.

## 2. Experimental Section

### 2.1. Materials

Refined soybean oil (SBO), hydrogen peroxide (H_2_O_2_, 30%), glacial acetic acid (99%), urea, acidic alumina (50–70 um), 1-thioglycerol, 1,4-phenylenebisboronic acid, and 4-dimethylaminopyridine (DMAP) were supplied by Adamas-beta (Shanghai, China). All other reagents were commercial chemicals and used as received without further purification.

### 2.2. Preparation of Epoxidized Soybean Oil (ESBO)

A mixture of 50 g SBO, 45 g H_2_O_2_ (30%), 12.5 g glacial acetic acid, 0.5 g urea, and 6 g acidic alumina was added into a flask equipped with a stir. The mixture was heated at 60 °C for 6 h to facilitate the epoxidation reaction. Glacial acetic acid and hydrogen peroxide were first catalyzed by acidic alumina to generate peroxyacetic acid, and then peroxyacetic acid diffused to the oil phase to react with unsaturated double bonds in SBO to form epoxy groups. Because the epoxy group is a ternary ring structure, it has good chemical activity, it is unstable under the action of acid and water, and it is easy to have ring opening side reactions. Therefore, urea was added as a stabilizer in this experiment to inhibit the epoxy ring-opening reaction, improve the reaction rate of epoxidation, and shorten the reaction time. After the epoxidation reaction was completed, the crude product contained glacial acetic acid and water which had to be removed. The solution was statically stratified, the lower waste liquid was separated, and the oil layer was washed with aqueous sodium hydroxide (0.1 mol/L) to pH = 5~6. The volume ratio of each dosage to oil is 1:1. If too much aqueous solution is added or the concentration is too high, it may cause the epoxide to open the ring, forming an emulsion and extending the delamination time. Moreover, adding a small amount of inorganic salt (1% NaCl) to the aqueous solution to increase the water phase density can shorten the delamination time, and the inorganic salt ions brought in the process can be removed in the next step of washing. Then, it was washed with deionized water to pH = 7.0. The residue water was removed under pressure at 80 °C to obtain epoxy soybean oil.

### 2.3. The Synthesis of 2,2′-(1,4-Phenylene)-bis(4-mercaptan-1,3,2-dioxaborolane) (BDB)

BDB was synthesized according to the previous report with a few modifications [38]. Briefly, 1-thioglycerol (10.8 g, 0.1 mol) and 1,4-phenylenebisboronic acid (8.3 g, 0.05 mol) were dissolved in ethanol (100 mL) and stirred for 24 h at room temperature. Finally, the solvent ethanol and the by-product water were removed under a reduced pressure to obtain the target compound as a white solid. The successful synthesis of BDB was explicitly confirmed.

### 2.4. Preparation of Cross-Linked Epoxy Resin (ESBO-B_x_)

Approximately 3 g ESBO and 1.96 g BDB cross-linker were dissolved in 30 mL DMAc, and then 0.05 g DMAP catalyst was added into the mixture. After being stirred for 5 min, the solution was cast into a PTFE mold (10 × 10 × 1 cm^3^) and placed in an oven for 4 d at 80 °C to obtain a cross-linked film. For convenience, the film was denoted as ESBO-B_1.0_ in which the number 1.0 represented the mole ratio of mercaptol groups and epoxy groups in the preparation. Therefore, other films, ESBO-B_0_._8_ and ESBO-B_0.6_, could be prepared according to a similar process.

### 2.5. Characterization

The chemical structures were characterized by a Bruker AV500 MHz spectrometer (Bruker BioSpin Corp., Billerica, MA, USA) at room temperature. ^1^H NMR chemical shifts were referenced to tetramethylsilane (TMS).

The mechanical properties of the prepared films were tested with an electronic universal testing machine (Instron 4465, Instron Engineering Corporation, Norwood, MA, USA) at a crosshead speed of 10 mm min^−1^. Dumbbell-shaped specimens were cut according to GB/T528 (overall length: 50 mm; inner width: 4 mm). At least five specimens per experimental point were tested in all mechanical measurements to obtain reliable values. The toughness was calculated according to the integral area of the stress–strain curve.

The swelling experiments were conducted as follows: a piece of film (m_0_ g) was immersed in DMAc (50 mL) for 3 d and then the swelling film was wiped softly by filter paper and weighted (m_1_ g). Lastly, the swelling film was dried in an oven at 80 °C to a constant weight (m_2_ g). The swelling ratio (SR) and gel content (GF) were calculated according to Equations (1) and (2), respectively.

(1) $$\mathrm{SR}=\frac{m_{1}-m_{2}}{m_{2}}$$

(2) $$\mathrm{GF}=\frac{m_{2}}{m_{0}}$$

Differential scanning calorimetry (DSC) analyses were performed on DSC 25 (TA Instruments, New Castle, DE, USA) under nitrogen atmosphere at a heating or cooling rate of 20 °C/min. The samples were heated from room temperature to 80 °C and then cooled down to −80 °C to eliminate the thermal history.

The self-healing ability was studied using an optical microscope (UPT200I, Proiser, Valencia, Spain). A piece of film was cracked using a knife and repaired at in oven at 90 °C. A glass slide was added to the sample to offer a small pressure.

The recycling experiments were conducted via a vulcanizer. The sample pieces were gathered and put into a steel mold on a press vulcanizer under 10 MPa at 120 °C for 60 min to form new samples. The sample dimension was 5 × 5 × 1 cm^3^. The process was repeated three times, and each recycled sample was tested using an electronic universal testing machine and differential scanning calorimetry.

The lap-shear tests were carried out on an electronic universal testing machine (Instron 4465). The glass sheets were 75 mm × 12.5 mm × 1 mm and cleaned by deionized water and ethanol before use. ESBO-B_1.0_ was cut and placed between the two glass sheets and the overlapping area was measured to be 12.5 mm × 12.5 mm with an interval thickness of 0.4 mm. After that, the overlapping area was heated at 120 °C under a pressure of 0.6 MPa for 30 min. The two glass interfaces were bonded together, and the original lap-shear sample was obtained. The followed lap-shear test was conducted to evaluate the adhesive strength. The re-assembled lap-shear sample was prepared as follows. The damaged interfaces of the original sample were re-contacted under the same conditions as when preparing the original sample. Tensile tests were also conducted.

## 3. Results and Discussion

**Figure 1:**
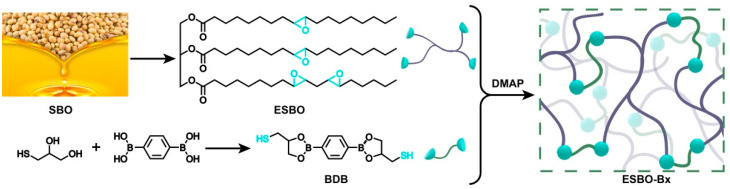
The synthetic route of cross-linked network ESBO-B_x_ based on the biomass-derived ESO and cross-linker BDB.

The primary component of SBO is a mixture of glycerol fatty acid esters, with the majority of its fatty acids being unsaturated. In the production of ESBO, the reaction involves unsaturated fatty acids that contain double bonds. This reaction can be divided into two steps. In the presence of catalysts, hydrogen peroxide reacts with acetic acid to produce peroxyacetic acid, which is used to convert the double bonds into epoxy groups. To achieve a cross-linked network, BDB is synthesized and utilized as the cross-linker, based on the reaction between the epoxy groups and mercaptol groups (as shown in Figure 1). The boronic esters are introduced as the dynamic component, contributing to the continuous construction of the network.

**Figure 2:**
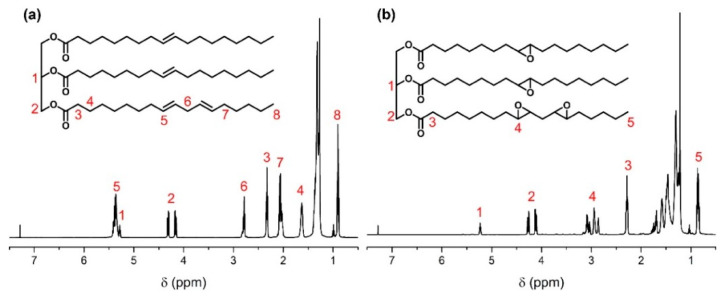
^1^H NMR spectra of (**a**) SBO and (**b**) ESBO.

The chemical structures of SBO and ESBO were characterized by ^1^H NMR. As shown in Figure 2a, the peaks at 5.3–5.4 ppm belong to unsaturated double bonds in SBO, while these peaks disappeared in the spectrum of ESBO. In comparison with SBO, the peaks at 2.8–3.2 ppm (Figure 2b) were attributed to the epoxy groups, which indicated the successful epoxidation reaction. In addition, it could be calculated based on the peak integrals that there are four epoxy groups on average of each triglyceride. 

**Figure 3:**
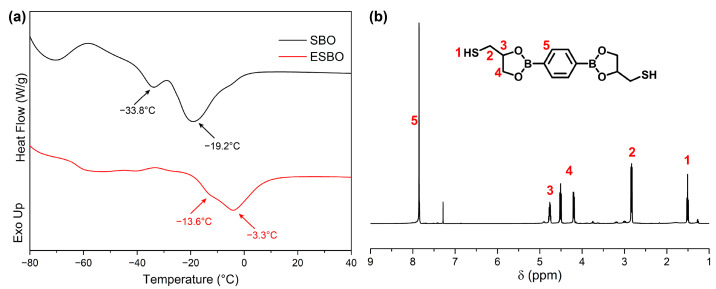
(**a**) DSC curves of SBO and ESO and (**b**) ^1^H NMR spectrum of BDB.

The DSC curves for SBO and ESBO are presented in Figure 3a. In the SBO curve, two distinct endothermic peaks were observed at approximately −33.8 °C and −19.2 °C, indicating the melting points of SBO at these temperatures. In contrast, ESBO exhibited a melting point at around −13.6 °C and −3.3 °C, suggesting that the functionalization of SBO with epoxide groups led to increased intermolecular interactions and subsequently raised the melting temperature. This shift in melting temperature further validated the successful synthesis of ESBO.

To construct the covalent cross-linked network and introduce dynamic boronic esters, the cross-linker BDB was synthesized, and the chemical structure was confirmed by ^1^H NMR as depicted in Figure 3b. The peaks around 1.5 ppm belonged to mercaptol groups. The peaks at 4.2 ppm, 4.5 ppm, 4.7 ppm, and 7.8 ppm demonstrated that boronic esters were obtained.

**Figure 4:**
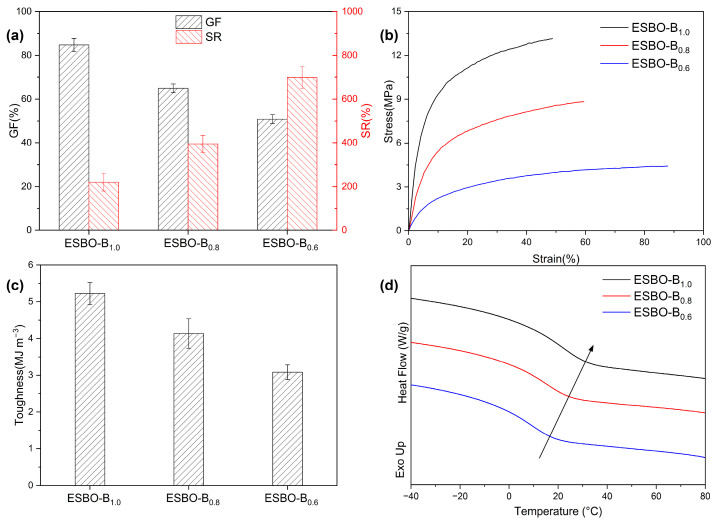
(**a**) The swelling experiment results, (**b**) stress–strain curves, (**c**) calculated toughness based on tensile tests, and (**d**) DSC curves of ESBO-B_x_. The error bars correspond to the standard deviation obtained from at least five samples.

According to the ^1^H NMR results mentioned earlier and the well-established reaction between epoxy groups and mercaptol groups, to verify the presence of the network, the resistance of the prepared films to solvents was evaluated using DMAc. The swelling ratio (SR) and gel fraction (GF) were analyzed and are presented in Figure 4a. The results showed that the GF and SR of ESBO-B_0.6_ were approximately 51% and 701%, respectively. However, with an increase in the amount of BDB used, the GF of ESBO-B_1.0_ increased to 84%. This indicated that a higher amount of BDB led to an increased cross-linking density and the improved solvent resistance of the resulting cross-linked network.

As is known, the properties of the adhesive are influenced by the number of cross-linkers, which is important for practical applications. Therefore, the obtained films were subjected to tensile tests, and the resulting curves are shown in Figure 4b. It was evident that the mechanical properties could be controlled by adjusting the amount of BDB used. For example, the stress at the break of ESBO-B_1.0_ was 13.15 MPa, which was higher than that of ESBO-B_0.6_ (4.42 MPa), due to the increased usage of BDB during the preparation process. The calculated toughness based on the integral area of the curves also confirmed that BDB strengthened the adhesive. As shown in Figure 4c, the toughness of ESBO-B_1.0_ is approximately 5.23 MJ m^−3^, whereas ESBO-B_0.6_ only has 3.01 MJ m^−3^. The tensile results indicated that the mechanical properties of the adhesive could be tailored to meet specific requirements. Additionally, based on the DSC results (Figure 4d), the glass transition temperature (*T*_g_) gradually increased with the amount of BDB. For instance, ESBO-B_0.6_ transitioned from a glass state to a rubber state at around 16.8 °C. In comparison, the *T*_g_ of ESBO-B_1.0_ was approximately 31.2 °C, as the interaction between molecular chains strengthened with the increased amount of BDB. 

**Figure 5:**
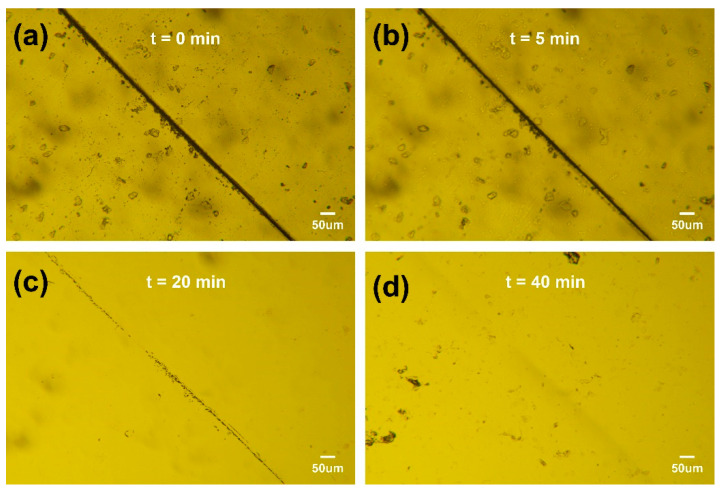
Optical microscope images of the crack healing of ESBO-B_1.0_ networks at 90 °C for (**a**) 0 min, (**b**) 5 min, (**c**) 20 min, (**d**) 40 min.

The deterioration of adhesive performance often stems from internal cracks. Due to the difficulty of the timely detection and repair of micro-cracks, the mechanical performance of materials is reduced, and their service life is shortened, limiting their application range. Therefore, the timely repair of cracks, especially the realization of self-healing of materials, is of great significance for improving the utilization efficiency of materials. The concept of self-healing originates from the healing of biological skin damage, which involves the release and polymerization of repair agents stored inside the material or the reformation of reversible interactions within the material. Self-healing materials can perceive changes in the external environment and respond appropriately, ultimately restoring their own performance. They are a widely applicable and urgently needed type of smart material. Self-healing materials can be divided into two categories. One is exogenous, which refers to filling the material with composite functional substances to achieve self-healing. Typical repair methods include microcapsule and hollow fiber methods, where the repair mechanism involves adding monomer-containing microcapsules to the material. When the material is damaged, the cracks propagate within the material, and the monomers in the microcapsules are released and come into contact with the catalyst in the material matrix, triggering polymerization and ultimately repairing the damage. However, this type of repair method has obvious limitations, as the repair agents encapsulated in the microcapsules are limited, and once the repair agents are depleted, the material loses its self-healing ability. The other one is intrinsic, which refers to providing energy to the material to enable covalent or non-covalent interactions within the material itself for self-healing. In the absence of external repair agents, reversible interactions are introduced into the polymer matrix, giving it dynamic characteristics and environmental responsiveness, thus achieving self-healing. Compared with exogenous self-healing materials, the biggest advantage of intrinsic self-healing materials is that they can theoretically achieve multiple self-healings. Therefore, ESBO-B_x_ should have the characteristics of intrinsic self-healing materials due to the incorporation of boronic esters. In order to examine its self-healing capability, ESBO-B_1.0_ was utilized as a representative case and intentionally scratched to create a surface crack. Subsequently, it was subjected to heating at 90 °C for varying durations. As depicted in Figure 5, the crack gradually diminished with the increasing heating time, demonstrating exceptional self-healing performance. This phenomenon could be attributed to the exchange of dynamic boronic esters, which led to the rearrangement of the cross-linked network. 

**Figure 6:**
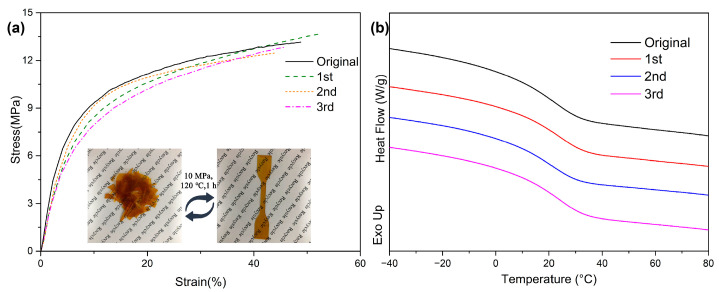
(**a**) Stress–strain curves and (**b**) DSC curves of the original ESBO-B_1.0_ and the recycled samples.

Moreover, the addition of dynamic boronic esters endowed ESBO-B_x_ with the ability to be recycled. As depicted in Figure 6a, a piece of ESBO-B_1.0_ film was fragmented and then subjected to hot pressing at 120 °C and 10 MPa for 1 h. Consequently, an intact film without apparent defects was obtained. This recycling process could be repeated up to three times, and the subsequent tensile tests were conducted to assess the efficiency of recycling. As demonstrated in Figure 6a, the stress–strain curve of ESBO-B_1.0_ after each cycle exhibited a similar pattern. After three cycles of hot pressing, the DSC tests revealed that the *T*_g_ did not undergo significant changes compared to the original sample, indicating that the crosslinking density remained constant while the network structure was adjusted (Figure 6b). The capability to retain properties in the recycled networks is attributed to the presence of abundant dynamic boronic esters, which facilitate the rearrangement of the network topology.

**Figure 7:**
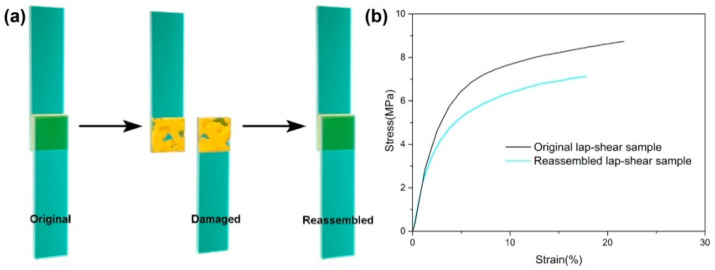
(**a**) Schematic view of re-assembled tests for ESBO- B_1.0_ networks. (**b**) Lap-shear strength of original and re-assembled samples.

After demonstrating the recycling and self-healing properties of ESBO-B_x_, its potential as an adhesive was investigated using the lap-shear test. To prepare the adhesion samples, two glass sheets were bonded with cured ESBO-B_1.0_, as shown in Figure 7a. The overlapping area measured 12.5 mm × 12.5 mm, with an interval thickness of 0.4 mm. After heating, the two interfaces were bonded together, and the lap-shear strength was tested, resulting in a value of 8.73 MPa (Figure 7b). Unlike traditional epoxy adhesives, the ESBO-B_1.0_ adhesive could be easily re-assembled due to the presence of dynamic boronic esters. Consequently, the failed halves of the adhesive were effortlessly rebonded upon heating at 120 °C for 30 min. The repaired lap-shear strength was measured at 7.11 MPa, with a stress recovery efficiency of approximately 81%. Importantly, adhesive residue was observed on the surfaces of both the original and repaired glass sheets after the tensile tests. This finding indicates that the lap-shear damage was caused by cohesive failure, rather than adhesive failure between the glass and adhesive interfaces.

## 4. Conclusions

In this study, SBO was modified and developed to create dynamic covalent networks that involved dynamic boronic esters. The successful modification of SBO and the synthesis of the cross-linker were confirmed by the ^1^H NMR results and DSC curves. Swelling experiments demonstrated the formation of cross-linked networks, which exhibited solvent resistance. The stress at break of the networks varied depending on the molar ratios between ESBO and BDB, ranging from 4.42 MPa to 13.15 Mpa. Additionally, the *T*_g_ of the networks, as evaluated by DSC, ranged from 16.8 °C to 31.2 °C. The self-healing and recycling experiments showed that the networks underwent topology rearrangement at elevated temperatures. The presence of polar groups and boronic esters allowed the networks to function as adhesives, facilitating reassembly when the bonded interface was broken. This recyclable adhesive, derived from biomass, possesses self-healing and re-assembly abilities, which contribute to reduced energy consumption and reduced environmental pollution.

## Data Availability

The data presented in this study are available upon request from the corresponding authors.
